# Supplementary material for: SARS-CoV-2 mRNA-vaccine candidate; COReNAPCIN®, induces robust humoral and cellular immunity in mice and non-human primates
Source: NPJ Vaccines. 2022 Sep 2;7:105. doi: 10.1038/s41541-022-00528-3 (PMC9438359; doi:10.1038/s41541-022-00528-3)
Supplement: Supplementary file 1 — Supplemental Material [file 41541_2022_528_MOESM1_ESM.pdf]

Supplementary Table 1 COReNAPCIN® Immunogenicity assessments summary

Test		BALB/C Mice	C57BL6 Mice	Rhesus Macaque
**Humoral immunity**				
Anti-Spike specific IgG binding antibody titer		✓	✓	✓
Anti-RBD IgG binding antibody titer		✓	✓	✓
cVNT		-	-	✓
pVNT		✓	✓	✓
sVNT(ACE2 inhibition)		✓	✓	✓
**Th1/Th2 balance**				
IgG1 /IgG2 ratio		✓	-	-
SARS-CoV-2 specific cytokines secretion by Splenocytes/PBMCs	IFN-γ	✓	✓	✓
	IL-4	✓	✓	✓
**Cellular immunity**				
SARS-CoV-2 Spike specific T cell populations (flow cytometry)		✓	✓	-
ELISPOT (SARS-CoV-2 Spike-specific IFN-γ-secreting Splenocytes/PBMCs)		-	-	✓
**Challenge study**				
2×10^8 PFU of SARS-CoV-2 Challenge		-	-	✓
Virus detection (PCR for detection of nasal and rectal RdRp and N genes)		-	-	✓
Frequency of PMNs in BAL (cell counting and differential analysis)		-	-	✓
Histopathology after challenge		-	-	✓

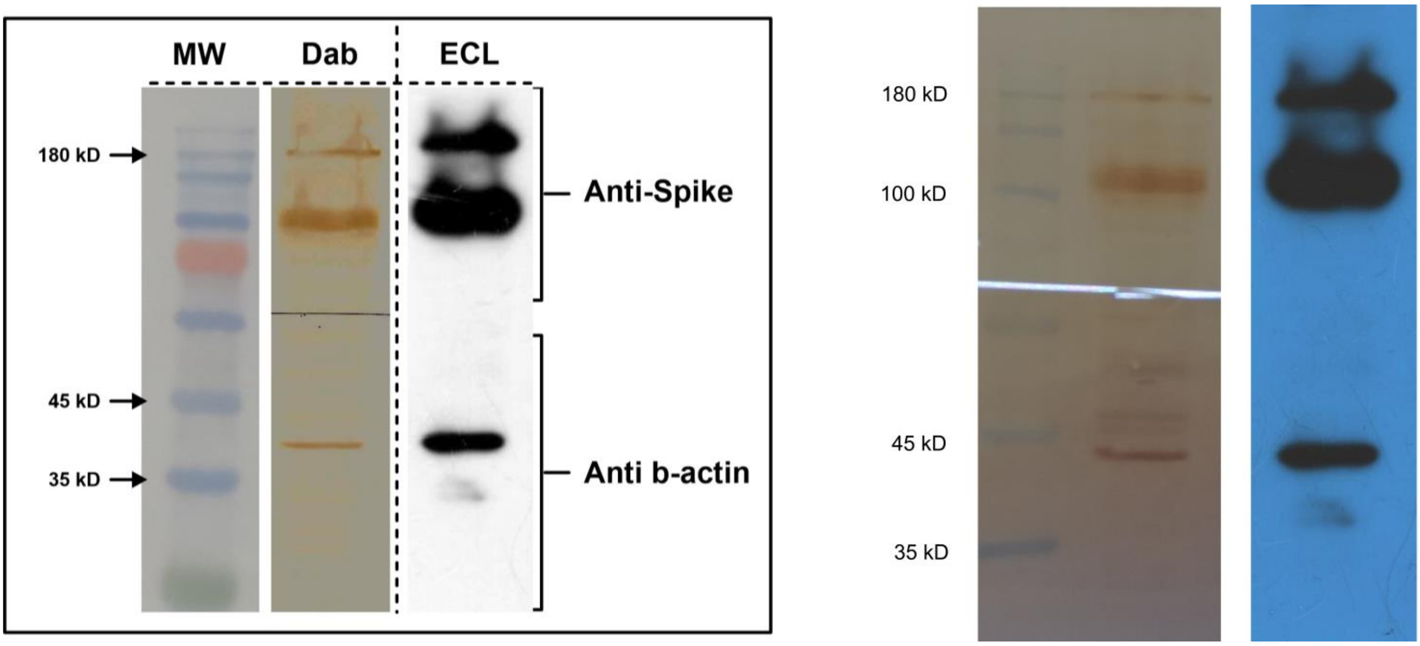

Supplementary Figure 1: Evaluation of SARS-CoV-2 Spike glycoprotein-2P expression by Western Blotting.

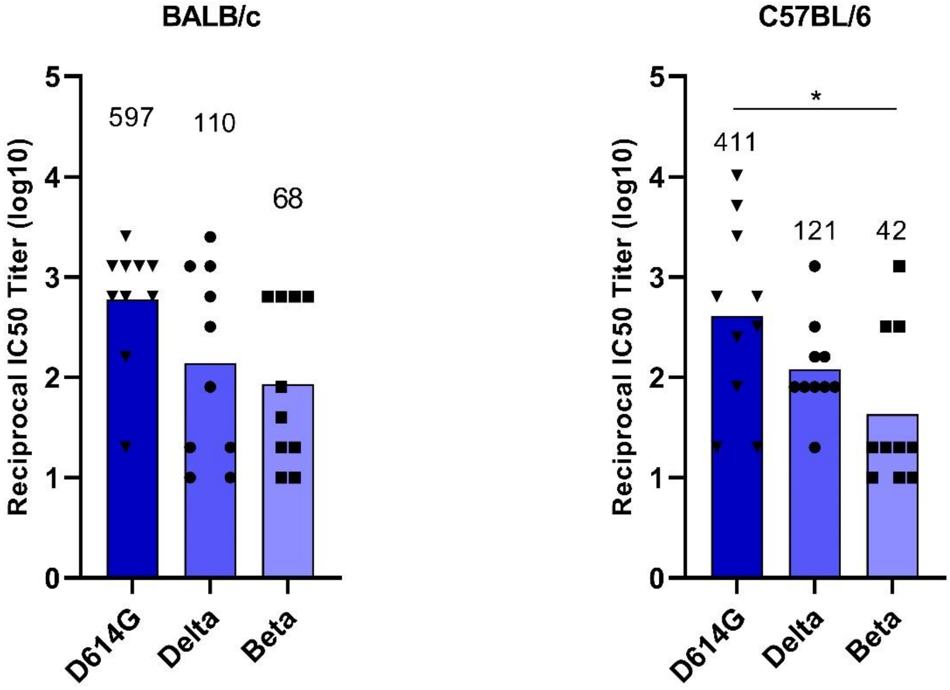

Supplementary Figure 2: Comparison of neutralizing activity against different variants in vaccinated mice. *BALB/c (left graph) (n=10 each group) and C57BL/6 mice (right graph) (n=10 each group) were immunized with 3 µg COReNAPCIN® at days 0 and 21. The sera samples collected at day 20 post booster dose, were assessed for their neutralizing capacity against SARS-CoV-2 D614G, Delta, and Beta pseudotyped viruses.*

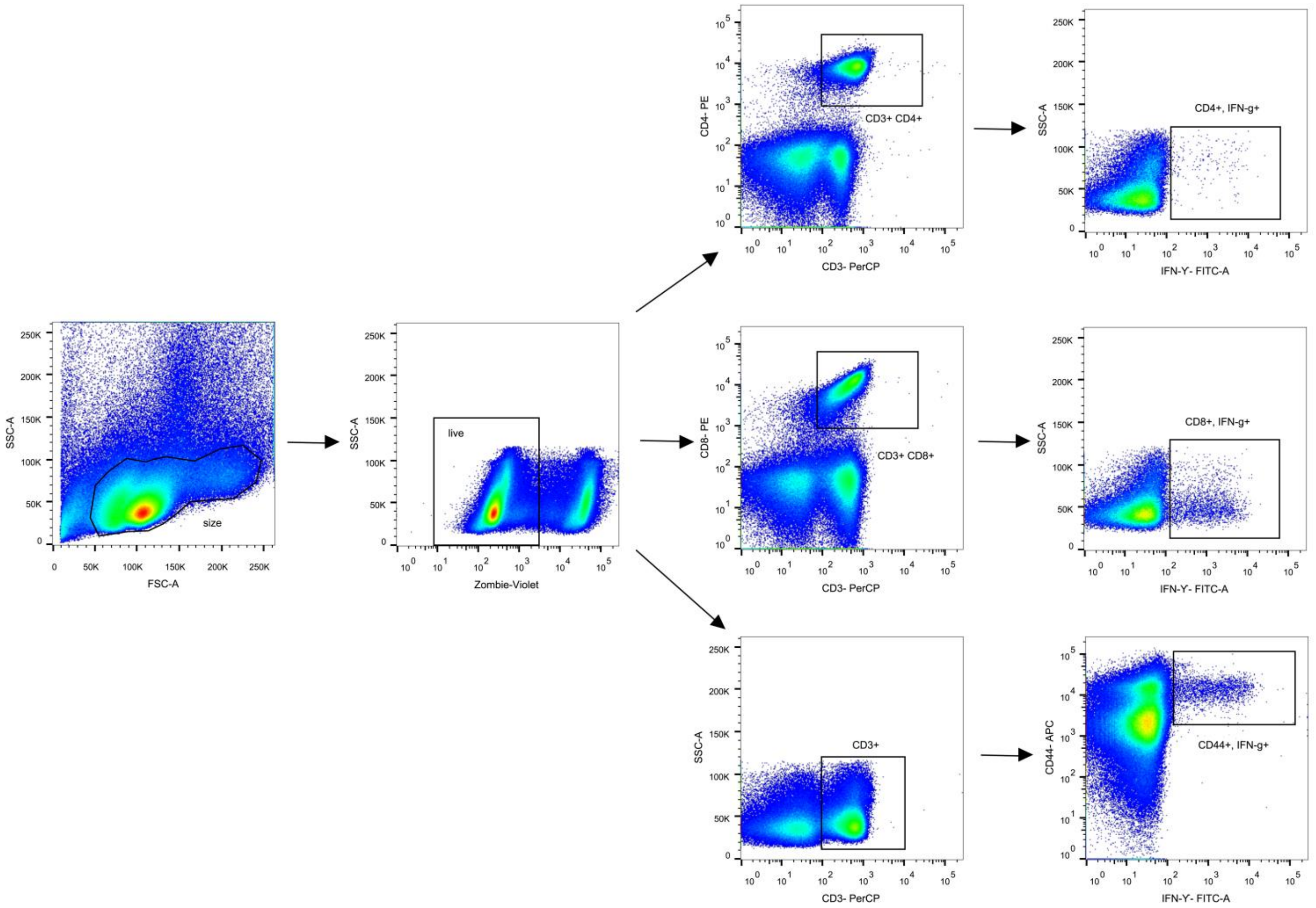

Supplementary Figure 3: Gating strategies for Selection of T cell population.

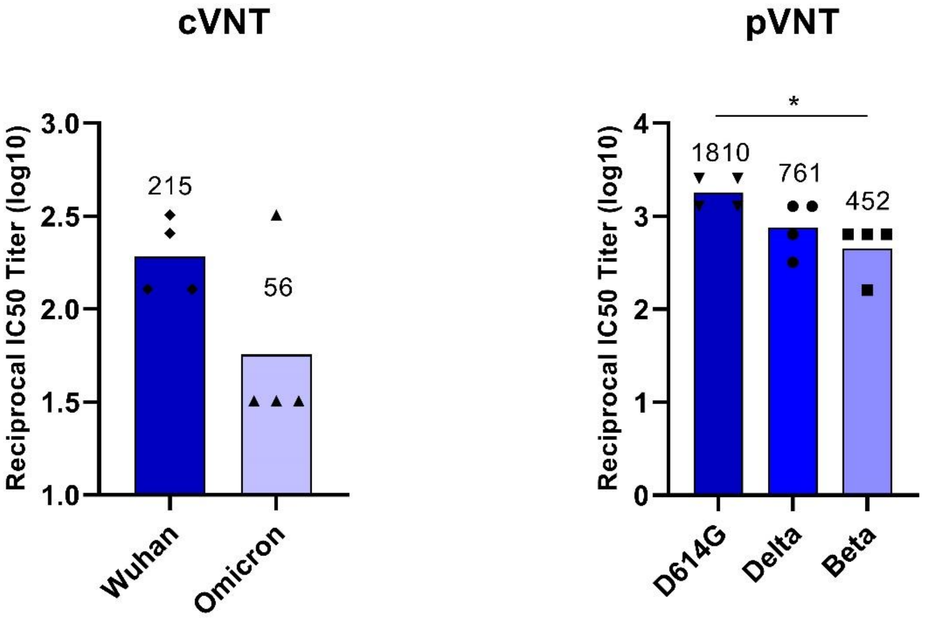

Supplementary Figure 4: Comparison of neutralizing activity against different variants in vaccinated macaques. *Rhesus macaques (n=4) were immunized with either 30 µg or 50 µg COReNAPCIN® at days 0 and 28 and their sera were collected at day 14 post booster dose. The sera samples from all vaccinated Rhesus macaques were assessed for neutralizing capacity against Wuhan and Omicron variants of SARS-CoV-2 using cVNT (left graph) and against D614G, Delta and Beta pseudoviruses using pVNT (right graph).*

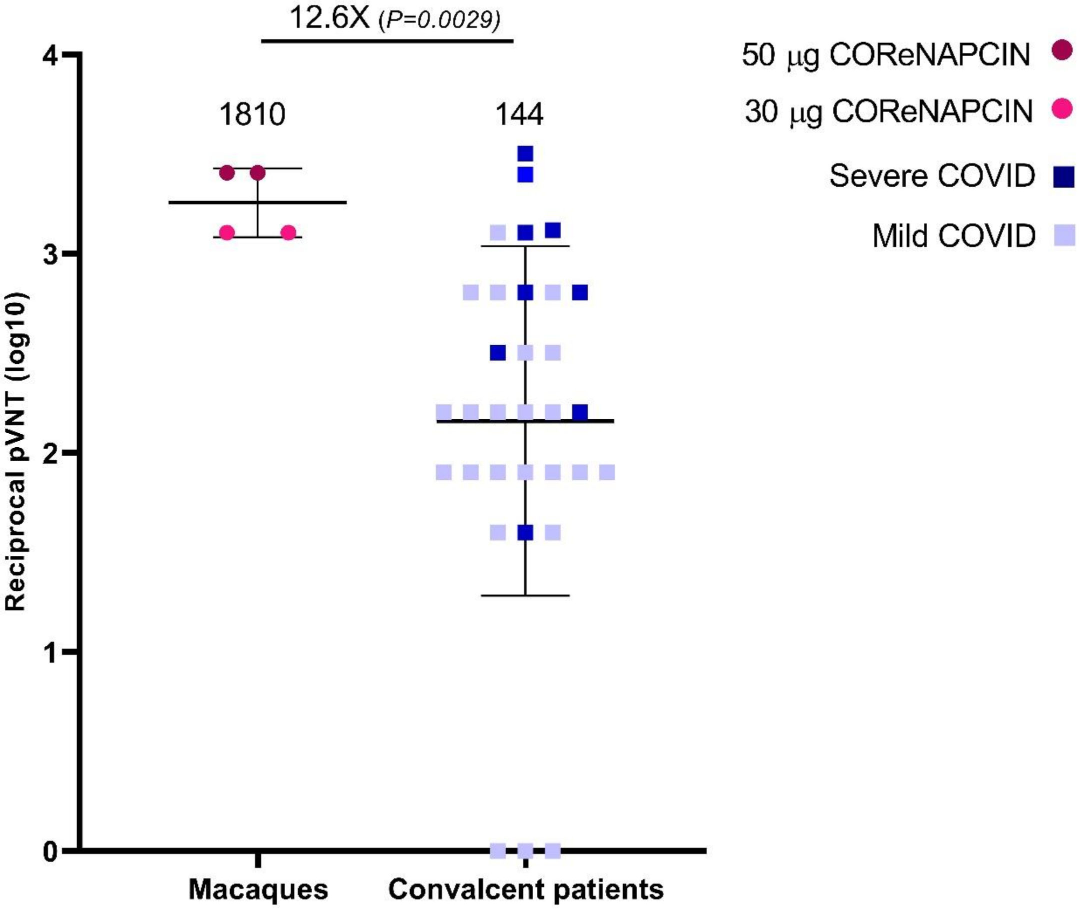

Supplementary Figure 5: Comparison of neutralizing activity against D614G in vaccinated macaques and convalescent patients. *Rhesus macaques (n=2 each group) were immunized with either 30 µg (pink) or 50 µg (purple) COReNAPCIN® at days 0 and 28 and their sera were collected at day 14 post after booster dose. Thirty two sera sample from convalescent patients were collected 14 days post SARS-CoV-2 infection (PCR positive date). The sera of COReNAPCIN® vaccinated Rhesus macaques (circle shape symbol, ●) and of human convalescent patients (square shape symbol, ■) were assessed for neutralizing antibodies against D614G variant using pVNT.*

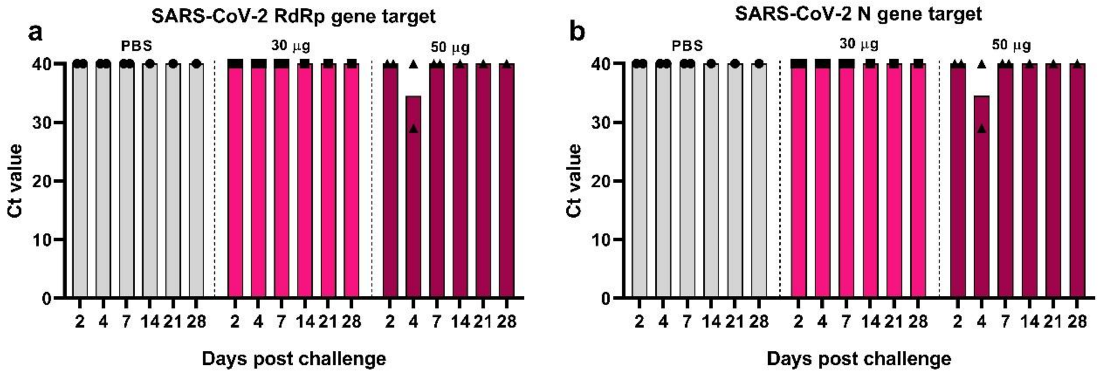

Supplementary Figure 6: Assessment of SARS-CoV-2 presence in rectal swabs. *Rectal swabs were collected at days 2, 4, 7, 14, 21, 28 post challenge, and assessed by RT-PCR for detection of RdRp (a) and N (b) genes of SARS-CoV-2.*
