# Supplementary material for: SARS-CoV-2 mRNA-vaccine candidate; COReNAPCIN®, induces robust humoral and cellular immunity in mice and non-human primates
Source: NPJ Vaccines. 2022 Sep 2;7:105. doi: 10.1038/s41541-022-00528-3 (PMC9438359; doi:10.1038/s41541-022-00528-3)
Supplement: Supplementary file 2 — Reporting Summary [file 41541_2022_528_MOESM2_ESM.pdf]

nature portfolio

Corresponding author(s): Vahid Khoddami

Last updated by author(s): May 1, 2022

Reporting Summary

Nature Portfolio wishes to improve the reproducibility of the work that we publish. This form provides structure for consistency and transparency in reporting. For further information on Nature Portfolio policies, see our Editorial Policies and the Editorial Policy Checklist.

Statistics

For all statistical analyses, confirm that the following items are present in the figure legend, table legend, main text, or Methods section.

n/a	Confirmed	
☐	☒	The exact sample size (*n*) for each experimental group/condition, given as a discrete number and unit of measurement
☒	☐	A statement on whether measurements were taken from distinct samples or whether the same sample was measured repeatedly
☐	☒	The statistical test(s) used AND whether they are one- or two-sided<br>*Only common tests should be described solely by name; describe more complex techniques in the Methods section.*
☒	☐	A description of all covariates tested
☒	☐	A description of any assumptions or corrections, such as tests of normality and adjustment for multiple comparisons
☒	☐	A full description of the statistical parameters including central tendency (e.g. means) or other basic estimates (e.g. regression coefficient) AND variation (e.g. standard deviation) or associated estimates of uncertainty (e.g. confidence intervals)
☒	☐	For null hypothesis testing, the test statistic (e.g. *F*, *t*, *r*) with confidence intervals, effect sizes, degrees of freedom and *P* value noted<br>*Give P values as exact values whenever suitable.*
☒	☐	For Bayesian analysis, information on the choice of priors and Markov chain Monte Carlo settings
☒	☐	For hierarchical and complex designs, identification of the appropriate level for tests and full reporting of outcomes
☒	☐	Estimates of effect sizes (e.g. Cohen's *d*, Pearson's *r*), indicating how they were calculated

Our web collection on statistics for biologists contains articles on many of the points above.

Software and code

Policy information about availability of computer code

Data collection	No software were used.
Data analysis	Flow jo, Graph pad prism, image J.

For manuscripts utilizing custom algorithms or software that are central to the research but not yet described in published literature, software must be made available to editors and reviewers. We strongly encourage code deposition in a community repository (e.g. GitHub). See the Nature Portfolio guidelines for submitting code & software for further information.

Data

Policy information about availability of data

All manuscripts must include a data availability statement. This statement should provide the following information, where applicable:
- Accession codes, unique identifiers, or web links for publicly available datasets
- A description of any restrictions on data availability
- For clinical datasets or third party data, please ensure that the statement adheres to our policy

The data that support the findings of this study are available on request from the corresponding author.

Field-specific reporting

Please select the one below that is the best fit for your research. If you are not sure, read the appropriate sections before making your selection.

☒ Life sciences ☐ Behavioural & social sciences ☐ Ecological, evolutionary & environmental sciences

For a reference copy of the document with all sections, see nature.com/documents/nr-reporting-summary-flat.pdf

Life sciences study design

All studies must disclose on these points even when the disclosure is negative.

Sample size	No sample size calculation was performed.
Data exclusions	No data excluded from study.
Replication	N.A for vaccination of animal models (for ethical concerns). but in immune response assessment (cellular or humeral) tests performed with several replication.
Randomization	all animal models were randomly distrusted into the treatment groups.
Blinding	N.A

Reporting for specific materials, systems and methods

We require information from authors about some types of materials, experimental systems and methods used in many studies. Here, indicate whether each material, system or method listed is relevant to your study. If you are not sure if a list item applies to your research, read the appropriate section before selecting a response.

Materials & experimental systems

n/a	Involved in the study	
☒	☐	Antibodies
☐	☒	Eukaryotic cell lines
☒	☐	Palaeontology and archaeology
☐	☒	Animals and other organisms
☒	☐	Human research participants
☒	☐	Clinical data
☒	☐	Dual use research of concern

Methods

n/a	Involved in the study	
☒	☐	ChIP-seq
☐	☒	Flow cytometry
☒	☐	MRI-based neuroimaging

Eukaryotic cell lines

Policy information about cell lines

Cell line source(s)	All cell lines were purchased from pasture institute of Iran.
Authentication	non of cell line used were Authenticated.
Mycoplasma contamination	All cell lines tested negative for Mycoplasma contamination.
Commonly misidentified lines (See ICLAC register)	N.A

Animals and other organisms

Policy information about studies involving animals; ARRIVE guidelines recommended for reporting animal research

Laboratory animals	6-8 weeks old BALB/c and C57BL6 mice, 1-2kg Rhesus macaques.
Wild animals	study did not involved wild animals.
Field-collected samples	study did not involved field collected samples.
Ethics oversight	All experiments involving animal, were conducted in accordance with the ethical regulations for the care and use of laboratory

Ethics oversight	animal and approved by Vice-Chancellor in Research Affairs-Tehran University of Medical Sciences with an ethic code of: IR.TUMS.VCR.REC1398.1055.

Note that full information on the approval of the study protocol must also be provided in the manuscript.

Flow Cytometry

Plots

Confirm that:

☒ The axis labels state the marker and fluorochrome used (e.g. CD4-FITC).

☒ The axis scales are clearly visible. Include numbers along axes only for bottom left plot of group (a 'group' is an analysis of identical markers).

☒ All plots are contour plots with outliers or pseudocolor plots.

☒ A numerical value for number of cells or percentage (with statistics) is provided.

Methodology

Sample preparation	Mice fresh splenocytes were separated by mechanical homogenization then filtered through a 70 µm cell strainer (Biologics). Red Blood Cells (RBCs) were removed by using RBC lysis buffer, then following wash with PBS, 106 splenocytes were ex vivo stimulated with SARS-CoV-2 peptide pool (JPT PM-WCPV-S-1) at concentration of 2 µg/ml of each peptide in RPMI (Biowest) supplemented with 10% heat inactivated FBS (Gibco). PMA/Ionomycin (BD Pharmingen™) and dimethyl sulfoxide (DMSO) (Sigma-Aldrich) were used as a positive and negative control, respectively. Brefeldin (Biolegend) as Golgi stop was added to each well one-hour post-incubation. Following eight hrs of incubation, the harvested cells, washed with PBS, and stained with Zombie Violet (Biolegend) at 1/200 dilution and incubated at room temperature for 30 min. After washing cells with staining buffer (PBS supplemented with 2% FBS and 0.05% NaN_3), anti CD3-Percp, anti CD8-PE, anti CD4-PE, anti CD44-APC (Biolegend) were used for cell surface staining followed by 20 min incubation at room temperature. For intracellular staining, cells were fixed and permeabilized with Cytofast fix/perm (Biolegend) according to the manufacturer instruction, then the anti IFN-γ-FITC (Biolegend) was added to the tubes. After 20 minutes incubation and washing with permeabilization and staining buffer (Biolegend), cells were acquired on BD FACS Lyrics and analyzed by Flowjo V10 (BD Biosciences).
Instrument	BD FACScalibur
Software	Flow Jo.
Cell population abundance	No cell sorting was performed.
Gating strategy	Positive and negative boundaries were set based on unstained and FMO controls. The detailed gating strategy for each population is depicted in supplementary fig. s2.

☒ Tick this box to confirm that a figure exemplifying the gating strategy is provided in the Supplementary Information.
